# Relentless Headaches and Syncope: A Case of Neuropsychiatric Systemic Lupus Erythematosus in a Pediatric Male Patient

**DOI:** 10.7759/cureus.78318

**Published:** 2025-01-31

**Authors:** Alaa S Mehair, Dalia Said, Najla Aljaberi

**Affiliations:** 1 Pediatric Medicine, Tawam Hospital, Abu Dhabi, ARE; 2 Pediatric Rheumatology, United Arab Emirates University, Abu Dhabi, ARE

**Keywords:** cerebral vasculitis, lupus nephritis, neuropsychiatric systemic lupus erythematosus (npsle), s: sle, s: systemic lupus erythematosus

## Abstract

Childhood systemic lupus erythematosus (cSLE) is a rare multisystem autoimmune disease with considerable morbidity as it has been typically reported to be more severe than in adults. Neuropsychiatric SLE (NPSLE) is the second leading cause of lupus-related morbidity in the pediatric age group, mostly occurring within the first year of onset, but may develop at any time. It can present with a range of vague specific neurologic manifestations which can be challenging to diagnose. Here, we present a case of NPSLE in an 11-year-old male, presenting less than a month after diagnosis with positive radiologic features.

## Introduction

Systemic lupus erythematosus (SLE) is a multisystem autoimmune disease with considerable morbidity [1]. The worldwide estimated prevalence of SLE is 3.3 to 9.7 per 100,000 children and adolescents [2]. Although the prevalence of SLE in the United Arab Emirates (UAE) is estimated to be relatively high based on the current scarce evidence [3-4], childhood SLE (cSLE) in the UAE is highly underreported and under-investigated. Among the systemic manifestations of SLE, neuropsychiatric SLE (NPSLE) is associated with pediatric presentations and high morbidity [5].

## Case presentation

An 11-year-old Arab male presented with a three-week history of intermittent fever, migratory joint pain, and swelling. His history was positive for fatigue and loss of appetite, but negative for skin rashes, mouth ulcers, weight loss, visual changes, or previous infections. On examination, he was pale, febrile, and had tachycardia with normal blood pressure. His weight was below the 3rd percentile. The musculoskeletal exam revealed an antalgic gait, with swelling and tenderness in both the metacarpophalangeal and tibiotarsal joints. Initial laboratory investigations showed a picture of autoimmune hemolytic anemia with leukopenia. An autoimmune rheumatologic disorder was suspected and further labs were requested. These revealed anemia, a positive Coombs test, and positive results for anti-nuclear antibodies (ANA), anti-double stranded DNA, anti-Smith, and anti-small nuclear ribonucleoprotein (anti-SM/RNP) (Table 1).

**Table 1:** Laboratory investigations as guided by the clinical presentation. - , not applicable; ANA, anti-nuclear antibody

Laboratory test	Value	Reference range
White blood cell count	3.2 x 10^9^/L	4.5-13.5 x 10^9^/L
Hemoglobin	72 g/L	11.7-16.6 g/L
Platelet count	232 x 10^9^/L	140-400 x 10^9^/L
Direct antigen test (Coombs)	Positive	-
Erythrocyte sedimentation rate	40 mm/hour	0-20 mm/hour
C-reactive protein	6.7 mg/L	<=5 mg/L
Anti-streptolysin O titer	74 IU/mL	<=240
Throat culture	Negative	-
Serum C3	0.17 g/L	0.9-1.8 g/L
Serum C4	0.03 g/L	0.10-0.40 g/L
ANA	Positive titer 1:1,280	-
Anti-double-stranded DNA	Positive titer >666.9 IU/mL	-
Anti-Smith	Positive	-
Anti-SM/RNP	Positive	-
Urine protein/creatinine ratio	2.52 g/g	-
24-hour urine protein	1.35 g/day	-
Urine analysis	No casts; red blood cells: 51 x 10^6^/L; white blood cells: 9 x 10^6^/L	-

A clinical diagnosis of SLE was made and the patient met the classification criteria of the 2019 European League Against Rheumatism/American College of Rheumatology (EULAR/ACR). The patient’s Systemic Lupus Erythematosus Disease Activity Index 2000 (SLEDAI-2K) scored 32, which indicated a very high activity of SLE. The patient was started on pulse steroids (Methylprednisolone IV) at 30 mg/kg for three days. Afterward, he was continued on oral prednisolone at 15 mg once daily as a starting dose. Lupus nephritis was suspected, and the patient underwent renal biopsy (after three doses of pulse steroids at 30 mg/kg). The biopsy showed diffuse proliferative lupus nephritis, classified as class IV-S (diffuse segmental) and G (diffuse global) of the International Society of Nephrology/Renal Pathology Society classification. Following that, the patient's oral prednisolone dose was increased to 20 mg daily, and mycophenolate mofetil (MMF) was started at 750 mg twice daily (BID), with a target dose of 600 mg/m² BID. As the patient developed hypertension and proteinuria, he was subsequently started on enalapril 5 mg twice daily. Cardiac echocardiogram (ECHO) and chest X-ray were normal. Further labs were sent to test for antiphospholipid antibodies, which revealed positive results for anti-cardiolipin IgG and anti-β2 glycoprotein IgG (Table 2).

**Table 2:** Antiphospholipid antibody and hypercoagulability investigations. - , not applicable; PT, prothrombin time; APTT, activated partial thromboplastin time; INR, international normalized ratio; CU, cubic units

Laboratory test	Value	Reference range
Anti-cardiolipin IgG	25.5 CU	<=20.0 CU
Anti-cardiolipin IgM	5.7 CU	<=20.0 CU
Anti-β2 glycoprotein IgG	41.7 CU	<=20.0 CU
Anti-β2 glycoprotein IgM	7.4 CU	<=20.0 CU
Lupus anticoagulant	Negative	-
PT	10.4 seconds	12.7-16.1 seconds
APTT	19.5 seconds	33.9-46.1 seconds
INR	0.94 seconds	0.97-1.30 seconds
D-Dimer	2.060 mg/L	0.129-0.523 mg/L

The patient was discharged home on oral prednisolone (20 mg) and MMF (750 mg BID). Ten days after diagnosis, he developed multiple intermittent episodes of headache, requiring two emergency room visits, where only supportive treatment was provided. Eventually, his headache became progressive, and he presented to the hospital again, this time experiencing two brief episodes of syncope.

His headache initially started in the frontal area, and within 24 hours, it started to radiate to the occipital region. The headache was mostly in the morning and associated with photophobia and early morning vomiting, which was non-projectile and non-bilious. On the day of the presentation, his pain was so intense that he lost consciousness for about two minutes and was unresponsive. He regained consciousness within two minutes. No abnormal jerky movements were noted.

Upon arrival at the emergency department, his vital signs were unstable and revealed elevated blood pressure of 155/115 mmHg, respiratory rate of 28 breaths per minute, and heart rate of 122 beats per minute. He was fully alert and conscious upon arrival at the hospital. His neurological and fundoscopic examinations were normal. Other systemic examinations were unremarkable, and both the ECHO and electrocardiogram (ECG) were normal. CT brain without contrast, which was done in the emergency department within one hour of the symptoms, ruled out cerebral hemorrhage, edema, or increased intracranial pressure.

**Figure 1:**
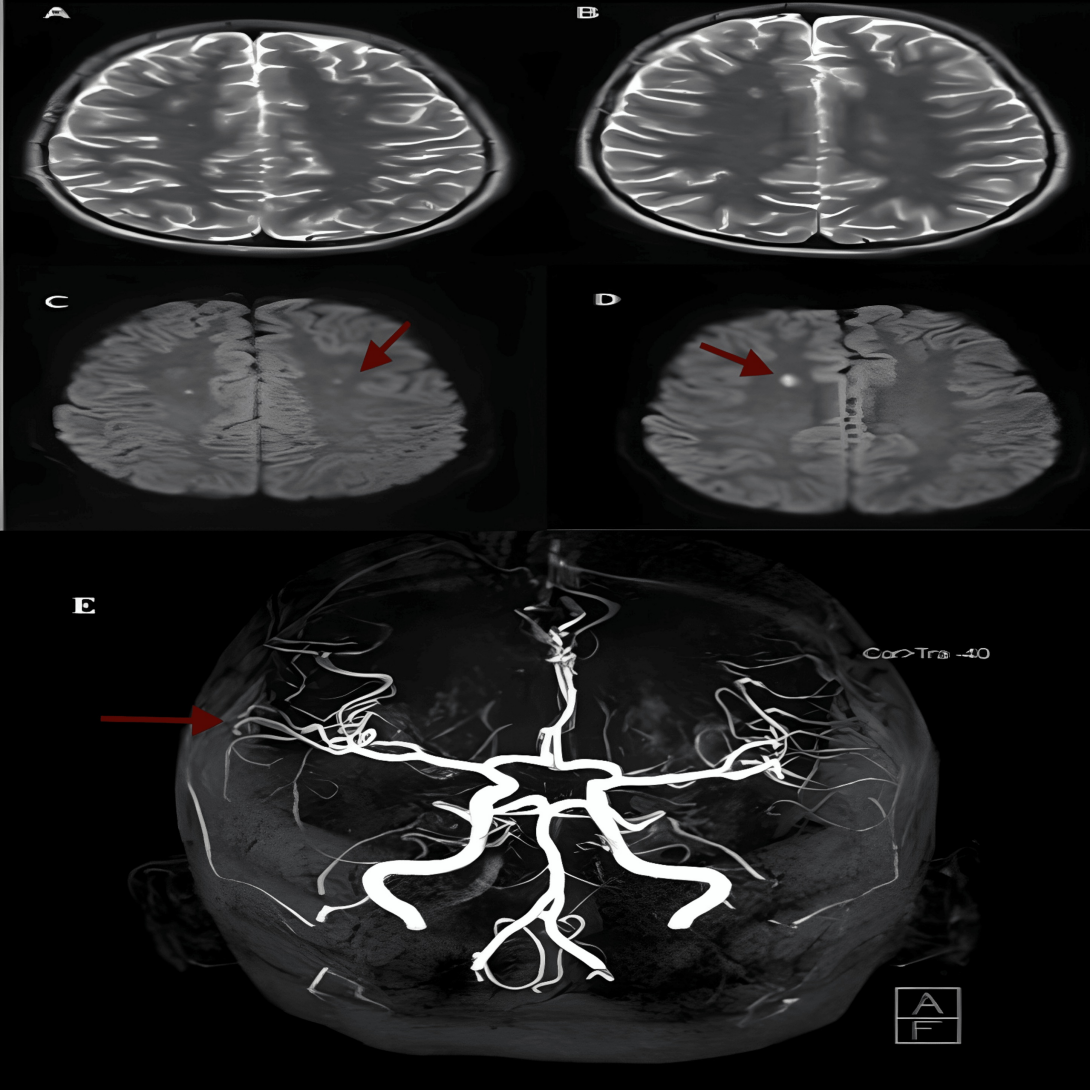
(A-D) MRI brain axial T2 sequences show high signal intensity foci involving both coronal radiata (bilateral centrum semiovale) (A) and the right frontal lobe (B), with corresponding restricted diffusion on axial diffusion-weighted images (DWI) (C, D), suggestive of neuropsychiatric SLE (arrows indicate the findings); (E) 3D TOF MR angiography in coronal reconstruction of the cerebral and cerebellar arteries demonstrates a normal appearance of the major arteries and their branches (arrow for reference). 3D TOF MR, three-dimensional time-of-flight magnetic resonance; SLE, systemic lupus erythematosus

In light of his hypertensive urgency, one dose of intravenous hydralazine 0.1 mg/ kg was given. A brain MRI with contrast showed small acute infarcts in the right frontal lobe for which he was diagnosed as a case of cerebral vasculitis as part of NPSLE manifestation. Magnetic resonance angiography (MRA) did not depict significant irregularities suggestive of underlying large-vessel vasculitis (Figure 1).

He was started on aspirin and received rituximab for active NPSLE. He received two doses of 375 mg/m^2^ of rituximab (amounting to 500 mg per dose).

The patient responded well to rituximab. Both his NPSLE and LN went into remission as he continued MMF and stopped steroids. Repeat antiphospholipid antibody testing continued to be negative afterward. He had a follow-up MRI brain after four months, which showed the presence of old right frontal focal infarction; otherwise, no significant abnormality was seen. No further neurologic issues were encountered as he had normal cognitive functioning and no limitations in daily life or school performance. This continued to be the case three years later at follow-up.

## Discussion

cSLE is typically more severe in presentation and morbidity than adult SLE [5]. The vast majority of childhood NPSLE cases present with central nervous system (CNS) involvement, with headaches occurring in up to 79% of cases [6]. The high female-to-male ratio of NPSLE was preserved in most pediatric studies [6] but was significantly more equal in one study from India [7]. There could be unique ethnic factors contributing to SLE presentations in male patients around the world. Patients with NPSLE typically present with an overall globally active disease, which should make the diagnosis of SLE easier. In many patients, especially those with renal involvement, posterior reversible encephalopathy syndrome could be suspected. However, it is always a reasonable approach to pursue imaging in patients with cSLE with neurologic signs, particularly at the beginning of their illness. Neuroimaging with MRI and, if needed, MRA is important to rule out focal findings such as acute vascular lesions, ischemia, and hemorrhage. However, non-specific white matter hyperintensities can be seen without correlation to the disease manifestations. White matter hyperintense lesions on MRI are the most encountered findings in patients with SLE. The correlation between these lesions and the neurological presentation is not entirely clear, as there are reports of these lesions in patients without overt NPSLE symptoms [8]. 

NPSLE can present with headaches, seizures, cerebrovascular events, cognitive dysfunction, and even psychosis, delirium, or depression. Stroke in SLE may be caused by different mechanisms, including antiphospholipid syndrome, cardiogenic embolism, intracranial hemorrhage secondary to hypertension, vasculopathy, and vasculitis. True vasculitis is difficult to detect on angiography unless large vessels become affected, which is rare [9].

## Conclusions

SLE is a multifactorial disease that ultimately leads to multisystem involvement. Despite being a rare entity, it is being increasingly recognized as a major pediatric rheumatologic disease. Furthermore, the great gender variation might have contributed to delayed diagnosis in male patients in a few case reports. However, it is very crucial to keep it in mind with such a clinical presentation as a more severe disease course is noted in male patients. 

A multidisciplinary approach is key to successful management, as controlling disease progression can be highly challenging and is essential for reducing morbidity and mortality. Management of NPSLE is directed toward controlling the disease and managing its symptoms. Various immunosuppressant medications are used, including MMF and azathioprine. In addition, rituximab is recommended as a biological therapy for managing similar cases. Further research is needed to identify the most effective treatments and improve control of NPSLE.
